# Composite dietary antioxidant index and risk of ischemic heart disease and stroke: insights from a UK Biobank large-scale cohort study

**DOI:** 10.3389/fnut.2026.1739431

**Published:** 2026-05-20

**Authors:** Shu-Ying Xu, Hao-Yu Liu, Li-Hong Chen, Xi-Xue Lu, Fang-Jie Hang, Lei-Yong Zhao

**Affiliations:** 1 Department of Acupuncture-Moxibustion, Tuina and Rehabilitation, Kunshan Hospital of Traditional Chinese Medicine, Kunshan Affiliated Hospital of Nanjing University of Chinese Medicine, Suzhou, China; 2 Department of Acupuncture, Affiliated Hospital of Traditional Chinese Medicine, Shandong First Medical University, Jinan, China; 3 Department of Acupuncture and Rehabilitation, Affiliated Hospital of Nanjing University of Chinese Medicine, Nanjing, Jiangsu, China

**Keywords:** association, CDAI, ICH, large-scale, stroke, UKB

## Abstract

**Background:**

Cardiovascular disease, particularly ischemic heart disease and stroke, remains a major global health burden. Although dietary antioxidants have been linked to cardiovascular health, previous studies have mainly focused on individual nutrients, and evidence regarding the combined effects of multiple antioxidants remains limited. This study aimed to examine the association between the composite dietary antioxidant index and the risks of ischemic heart disease and stroke in the UK Biobank cohort.

**Methods:**

This prospective cohort study included 66,382 participants from the UK Biobank. Data were derived from the UK Biobank prospective cohort. Cox proportional hazards models were applied to estimate hazard ratios (HRs) for IHD and stroke across quartiles of CDAI. Potential nonlinear associations were evaluated using restricted cubic spline models. In addition, the interaction analyses were conducted to explore the subgroup differences.

**Results:**

A total of 66,382 participants were included in the analysis, with 4,844 cases of IHD and 1,982 cases of stroke documented during a median follow-up of approximately 12.1 years for IHD and 12.4 years for stroke. Individuals with higher CDAI values demonstrated a trend of initially decreasing and then increasing risk of IHD and stroke compared with those in the lowest quartile. Nonlinear analyses revealed an inflection point at −0.30 for IHD and −0.29 for stroke. Below these thresholds, CDAI was inversely associated with disease risk, with hazard ratios (HRs) of 0.89 (95% CI: 0.81–0.97) for IHD and 0.82 (95% CI: 0.72–0.95) for stroke.

**Conclusion:**

This large-scale cohort study revealed that elevated CDAI within a specific range was associated with a reduced risk of IHD and stroke. These results underscore the relevance of antioxidant-rich dietary patterns for cardiovascular disease prevention.

## Introduction

Cardiovascular disease (CVD) is the leading cause of morbidity and mortality worldwide, with ischemic heart disease (IHD) and stroke constituting the two most prevalent subtypes (1). Together, these conditions contribute to enormous clinical and socioeconomic burdens. These burdens highlight the need for preventive strategies targeting upstream and modifiable determinants of cardiovascular health. Among these, diet represents a key target, as population-wide improvements in dietary quality have the potential to substantially reduce disease burden and improve long-term population health outcomes.

Oxidative stress and systemic inflammation are recognized as central mechanisms in the development of atherosclerosis and vascular injury (2). Endothelial dysfunction is also closely involved in these processes and contributes to cardiovascular disease progression (3, 4). Dietary factors play a critical role in modulating redox balance, and insufficient intake of antioxidant-rich foods has been linked to endothelial dysfunction, lipid peroxidation, and increased cardiovascular risk (5). At the same time, evidence at the nutrient level remains mixed. Although several observational studies and recent meta-analyses have reported associations between dietary antioxidant exposure and cardiovascular outcomes, findings from studies focusing on individual nutrients remain inconsistent (6–11). Emerging evidence from population-based studies suggests that the relationship between antioxidant intake and cardiovascular outcomes may be nonlinear, with potential threshold or saturation effects (12, 13). These inconsistencies may arise because single-nutrient analyses fail to capture synergistic dietary effects. Therefore, whole-diet approaches and composite antioxidant indicators may better reflect overall dietary antioxidant exposure than isolated nutrient assessments.

To address this limitation, the composite dietary antioxidant index (CDAI) has been proposed as an integrative measure reflecting the combined intake of major antioxidant micronutrients, including carotene, selenium, zinc, and vitamins A, C, and E (14, 15). Previous research has applied CDAI to evaluate associations with multiple health outcomes, such as cancer incidence, metabolic diseases, and all-cause mortality (16, 17). Collectively, these findings support CDAI as a useful indicator of overall dietary antioxidant exposure. However, evidence specifically focusing on cardiovascular outcomes remains limited and heterogeneous, particularly regarding long-term prospective associations. Multiple investigations have explored the link between the composite dietary antioxidant index (CDAI) and various cardiovascular outcomes. In a study based on a large U. S. population, Teng and colleagues identified an inverse nonlinear association between CDAI levels and the risk of stroke (12). Lin et al. also revealed an inverse and non-linear relationship between CDAI and ASCVD in adults (13). Moreover, Hu et al. suggested that higher levels of dietary antioxidants are associated with a reduced risk of both all-cause and cardiovascular-cause mortality in patients with CVD (18). Nevertheless, these investigations were either cross-sectional in design, restricted to specific populations, or limited by relatively short follow-up, thereby constraining their ability to establish causal inferences and generalize findings.

The UK Biobank (UKB), with its exceptionally large sample size, standardized dietary and lifestyle assessments, and long-term follow-up through linkage to hospital and mortality records, provides an unparalleled resource to overcome these limitations. Leveraging this cohort, the present study aimed to clarify the associations between CDAI and incident IHD and stroke, with particular attention to potential nonlinear relationships. Our findings may provide additional evidence for dietary antioxidant assessment and contribute to public health strategies emphasizing antioxidant-rich dietary patterns as part of cardiovascular prevention.

## Methods

### Study design and population

This was a prospective cohort study based on the UK Biobank database. Between 2006 and 2010, over 500,000 participants aged 40–69 years were recruited from 22 assessment centers across England, Scotland, and Wales. At baseline, participants provided informed consent, completed standardized touchscreen questionnaires and nurse-led interviews on sociodemographic characteristics, lifestyle factors, and medical history, underwent physical measurements, and donated biological samples (19). Health outcomes were ascertained prospectively through linkage with national electronic health records, including hospital admission data and death registries. Comprehensive details of the study design and data collection are publicly available on the UK Biobank website^1^ and have been described in detail elsewhere. Ethical approval for UK Biobank was granted by the North West Multi-Centre Research Ethics Committee (MREC), and all participants provided written informed consent prior to enrolment.

**Figure 1:**
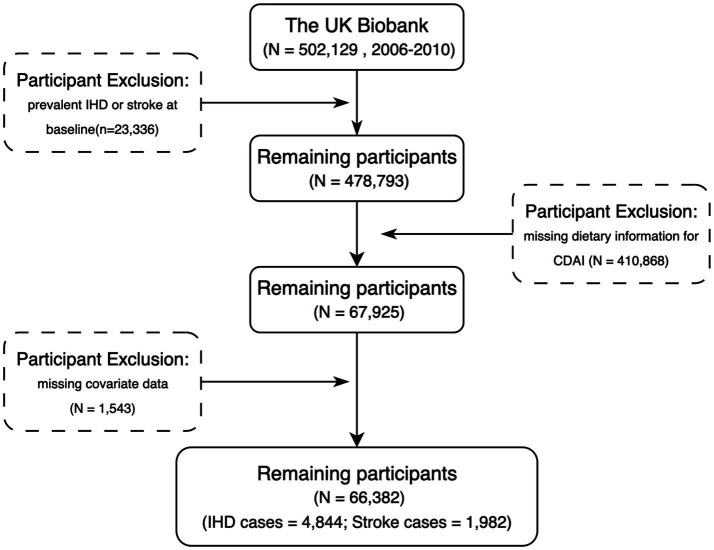
Flow chart of sample selection from UK Biobank.

The following exclusion criteria were applied: (i) participants with a history of ischemic heart disease (IHD) or stroke at baseline (*n* = 23,336); (ii) participants with missing dietary information required to calculate the composite dietary antioxidant index (CDAI) (*n* = 410,868); and (iii) participants with incomplete covariate data (*n* = 1,543). After these exclusions, 66,382 individuals were retained for the final analysis, among whom 4,844 incident IHD cases and 1,982 incident stroke cases were identified during follow-up (Figure 1).

### CDAI calculation

Dietary data were obtained in the UK Biobank using the Oxford WebQ, an online 24-h dietary recall questionnaire that has been validated in large-scale population studies. Participants completed the WebQ at baseline and, for a subset, at repeated follow-up visits. For those with multiple recalls, mean intakes across all available assessments were used to minimize within-person variability and to approximate usual dietary intake. The Oxford WebQ captures more than 200 food and beverage items, which are then linked to the UK Nutrient Databank for nutrient composition (20).

For the present analysis, six dietary antioxidants were selected based on their established biological activity and prior applications of the composite dietary antioxidant index. These included carotene, selenium, zinc, vitamin A, vitamin C, and vitamin E. For each nutrient, standardized values were calculated as follows: (individual intake − cohort mean intake) / standard deviation. The CDAI was constructed by summing the standardized values of the six nutrients, resulting in a continuous score, with higher values representing greater overall dietary antioxidant exposure. Nutrient intakes were not energy-adjusted in the present analysis. For primary analyses, CDAI was modeled both as a continuous variable (per one standard deviation increment) and in quartiles based on the population distribution. Quartile cut-points were derived directly from the analytic cohort (Q1 representing the lowest antioxidant intake and Q4 the highest). This dual approach allowed estimation of linear dose–response relationships as well as potential threshold effects.

Importantly, dietary supplement intake was not included in the construction of CDAI, as quantitative dose information for supplements is not systematically available in the UK Biobank. Thus, the CDAI reflects antioxidant exposure solely from foods and beverages. Participants with missing dietary recalls or incomplete nutrient information required for CDAI calculation were excluded from the analytic sample, as indicated in the flowchart.

This procedure ensures that the CDAI reflects the combined biological effects of multiple antioxidant nutrients rather than focusing on a single compound in isolation. Such an approach provides a more comprehensive assessment of dietary antioxidant exposure and its potential association with long-term cardiovascular outcomes.

### Assessment of outcomes

Incident ischemic heart disease cases within the UK Biobank cohort were ascertained through linkage to hospital inpatient records and death registries in England, Scotland, and Wales. IHD was defined according to the 10th Revision of the International Classification of Diseases (ICD-10) codes “I20–I25.” The date of the first hospital admission or death record carrying an eligible code was taken as the date of diagnosis. Stroke cases were identified via linkage to hospital admission data and national death registries across England, Scotland, and Wales. Stroke was classified based on ICD-10 codes “I60–I69.” The earliest date of hospital admission or death registration with a qualifying code was defined as the date of diagnosis.

### Covariates

Baseline covariates were collected at the initial assessment via touchscreen questionnaires, physical measurements, and health records. Age (years) was treated as a continuous variable, summarized as mean (SD). Sex was categorized as male or female. Ethnicity was dichotomized into White and non-White. Lifestyle behaviors included drinking status, smoking status, and physical activity. Drinking status was classified into never, previous, and current drinkers. Smoking status was grouped into never smokers, previous smokers, and current smokers. Physical activity was categorized as yes (active) or no (inactive) based on self-reported engagement in moderate-to-vigorous activities. Anthropometric measures included body mass index (BMI, kg/m^2^), calculated from standardized measurements of height and weight at baseline. Clinical conditions included self-reported and hospital record–verified history of hypertension, diabetes, and hyperlipidemia. Socioeconomic status was assessed using the Townsend deprivation index, with higher values indicating greater deprivation. These covariates were chosen based on prior knowledge of cardiovascular risk factors and their potential to confound the associations between the composite dietary antioxidant index (CDAI) and incident ischemic heart disease or stroke.

### Statistical analyses

The baseline profiles of participants were categorized by CDAI quartiles. Continuous data were expressed as mean values with corresponding standard deviations (SD), whereas categorical data were described by frequencies and proportions. Comparisons among the quartile groups were performed using one-way analysis of variance for continuous measures and the chi-square test for categorical measures. Associations of the CDAI with the incidence of IHD and stroke were analyzed through Cox proportional hazards models. The proportional hazards assumption was assessed using Schoenfeld residuals, and no substantial violations were observed. Person-years were calculated from the date of recruitment until the date of incident IHD or stroke, death, loss to follow-up, or the end of follow-up, whichever came first. CDAI was analyzed both as a continuous variable (per one SD increase) and as quartiles, with the lowest quartile serving as the reference group. Three models were constructed: Model 1 adjusted for age, sex, and ethnicity; Model 2 further adjusted for socioeconomic status, smoking, drinking, physical activity, body mass index, and dietary energy intake; and Model 3 additionally adjusted for baseline comorbidities, including hypertension, diabetes, and hyperlipidemia. Model 3 was constructed to assess the robustness of the associations after additional adjustment for variables that may partially act as mediators. Therefore, estimates from this model should be interpreted as conservative. Potential non-linear associations between CDAI and outcomes were explored using restricted cubic spline functions. The competing risk model was used to examine the risk of outcomes occurring in different CDAI groups over time. The interaction analyses were conducted to explore the subgroup differences. Given the exploratory nature of subgroup and interaction analyses, no formal adjustment for multiple comparisons was applied. Therefore, these results should be interpreted with caution. All statistical tests were two-sided, and a *p* value <0.05 was considered statistically significant. Analyses were performed using R software version 4.2.2 (R Foundation for Statistical Computing, Vienna, Austria).

## Results

### Baseline characteristics

Baseline characteristics of the 66,382 included participants are presented in Table 1. The mean age was 55.98 years (SD 8.17), and 43.74% were males. Participants with higher CDAI demonstrated generally healthier lifestyle profiles and more favorable cardiometabolic characteristics. Specifically, those in the highest quartile were less likely to be current smokers, more physically active, and had higher educational attainment compared with those in the lowest quartile. Dietary fiber intake also increased across CDAI quartiles, while inflammatory markers such as CRP showed modestly more favorable profiles at higher CDAI levels. The mean follow-up was 12.1 years (SD 2.3) for ischemic heart disease (IHD) and 12.4 years (SD 1.7) for stroke, during which 4,844 IHD and 1,982 stroke cases were documented.

**Table 1:** Baseline characteristics of participants.

Characteristic	Total *N* = 66,382	Q1 *N* = 16,596^1^	Q2 *N* = 16,595^1^	Q3 *N* = 16,595^1^	Q4 *N* = 16,596^1^	*p*-value
Age (years)						<0.001
Mean (SD)	55.98 (8.17)	55.41 (8.18)	56.05 (8.09)	56.31 (8.08)	56.14 (8.28)	
Gender						0.2
Female	37,347 (56.26%)	9,345 (56.31%)	9,244 (55.70%)	9,324 (56.19%)	9,434 (56.85%)	
Male	29,035 (43.74%)	7,251 (43.69%)	7,351 (44.30%)	7,271 (43.81%)	7,162 (43.15%)	
Ethnicity						<0.001
Non-White	4,026 (6.06%)	1,390 (8.38%)	888 (5.35%)	758 (4.57%)	990 (5.97%)	
White	62,356 (93.94%)	15,206 (91.62%)	15,707 (94.65%)	15,837 (95.43%)	15,606 (94.03%)	
Drinking status						<0.001
Never	2,441 (3.68%)	710 (4.28%)	582 (3.51%)	529 (3.19%)	620 (3.74%)	
Previous	2,190 (3.30%)	606 (3.65%)	502 (3.03%)	490 (2.95%)	592 (3.57%)	
Current	61,751 (93.02%)	15,280 (92.07%)	15,511 (93.47%)	15,576 (93.86%)	15,384 (92.70%)	
Smoking status						<0.001
Never	37,919 (57.12%)	9,158 (55.18%)	9,592 (57.80%)	9,614 (57.93%)	9,555 (57.57%)	
Previous	22,918 (34.52%)	5,607 (33.79%)	5,700 (34.35%)	5,755 (34.68%)	5,856 (35.29%)	
Current	5,545 (8.35%)	1,831 (11.03%)	1,303 (7.85%)	1,226 (7.39%)	1,185 (7.14%)	
Physical activity						<0.001
No	24,272 (36.56%)	6,619 (39.88%)	6,351 (38.27%)	5,995 (36.13%)	5,307 (31.98%)	
Yes	42,110 (63.44%)	9,977 (60.12%)	10,244 (61.73%)	10,600 (63.87%)	11,289 (68.02%)	
Education						<0.001
Unknown	6,663 (10.04%)	2,223 (13.39%)	1,621 (9.77%)	1,390 (8.38%)	1,429 (8.61%)	
College	26,136 (39.37%)	5,470 (32.96%)	6,574 (39.61%)	6,971 (42.01%)	7,121 (42.91%)	
Other levels	33,583 (50.59%)	8,903 (53.65%)	8,400 (50.62%)	8,234 (49.62%)	8,046 (48.48%)	
BMI						<0.001
< 30	51,709 (77.90%)	12,713 (76.60%)	12,992 (78.29%)	13,104 (78.96%)	12,900 (77.73%)	
≥30	14,673 (22.10%)	3,883 (23.40%)	3,603 (21.71%)	3,491 (21.04%)	3,696 (22.27%)	
TDI						<0.001
Median (Q1, Q3)	−1.94 (−3.48, 0.43)	−1.77 (−3.38, 0.81)	−2 (−3.51, 0.30)	−2.07 (−3.55, 0.18)	−1.89 (−3.44, 0.48)	
Diet score						<0.001
Median (Q1, Q3)	3 (2.00, 4.00)	3 (2.00, 3.00)	3 (2.00, 4.00)	3 (2.00, 4.00)	3 (2.00, 4.00)	
Sleep score						0.001
Median (Q1, Q3)	1 (1.00, 2.00)	1 (1.00, 2.00)	1 (1.00, 2.00)	1 (1.00, 2.00)	1 (1.00, 2.00)	
CRP (mg/L)						<0.001
Median (Q1, Q3)	1.21 (0.60, 2.51)	1.34 (0.64, 2.78)	1.20 (0.60, 2.51)	1.17 (0.59, 2.37)	1.16 (0.58, 2.37)	
Glucose (mmol/L)						0.5
Mean (SD)	5.11 (1.21)	5.16 (1.06)	5.15 (1.00)	5.16 (1.06)	5.16 (1.04)	
Hba1c (mmol/mol)						0.009
Mean (SD)	35.71 (6.16)	35.75 (6.29)	35.59 (6.08)	35.69 (6.07)	35.81 (6.20)	
Cholesterol (mmol/L)						0.010
Mean (SD)	5.74 (1.11)	5.74 (1.13)	5.75 (1.11)	5.76 (1.10)	5.72 (1.11)	
TG (mmol/L)						0.12
Median (Q1, Q3)	1.43 (1.01, 2.05)	1.42 (1.01, 2.05)	1.42 (1.01, 2.03)	1.43 (1.02, 2.06)	1.43 (1.01, 2.05)	
B12 intake (ug)						<0.001
Median (Q1, Q3)	5.25 (3.57, 7.54)	3.63 (2.47, 4.99)	4.96 (3.58, 6.65)	5.87 (4.26, 7.90)	7.41 (5.17, 10.60)	
Fibre intake (g)						<0.001
Median (Q1, Q3)	18.08 (7.93)	11.58 (4.88)	16.11 (5.03)	19.33 (5.54)	25.29 (8.48)	
Hypertension						<0.001
No	62,523 (94.19%)	15,528 (93.56%)	15,686 (94.52%)	15,676 (94.46%)	15,633 (94.20%)	
Yes	3,859 (5.81%)	1,068 (6.44%)	909 (5.48%)	919 (5.54%)	963 (5.80%)	
Hyperlipidemia status						0.008
No	65,346 (98.44%)	16,292 (98.17%)	16,337 (98.45%)	16,356 (98.56%)	16,361 (98.58%)	
Yes	1,036 (1.56%)	304 (1.83%)	258 (1.55%)	239 (1.44%)	235 (1.42%)	
DM						<0.001
No	65,514 (98.69%)	16,320 (98.34%)	16,415 (98.92%)	16,394 (98.79%)	16,385 (98.73%)	
Yes	868 (1.31%)	276.00 (1.66%)	180.00 (1.08%)	201.00 (1.21%)	211.00 (1.27%)	
IHD						<0.001
No	61,538 (92.70%)	15,298 (92.18%)	15,396 (92.77%)	15,491 (93.35%)	15,353 (92.51%)	
Yes	4,844 (7.30%)	1,298 (7.82%)	1,199 (7.23%)	1,104 (6.65%)	1,243 (7.49%)	
Stroke						<0.001
No	64,400 (97.01%)	16,041 (96.66%)	16,146 (97.29%)	16,111 (97.08%)	16,102 (97.02%)	
Yes	1,982 (2.99%)	555 (3.34%)	449 (2.71%)	484 (2.92%)	494 (2.98%)	

^1^n (%).

^2^Kruskal-Wallis rank sum test; Pearson’s Chi-squared test.

### Association between CDAI and ischemic heart disease

**Figure 2:**
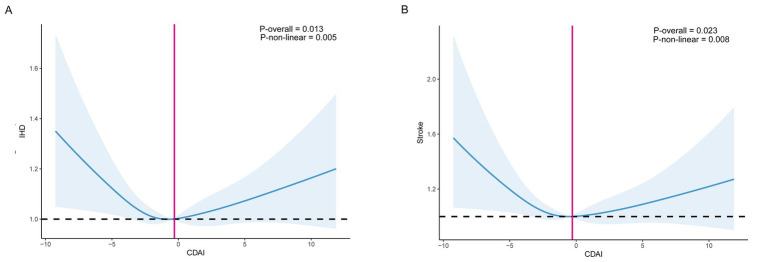
Association of composite dietary antioxidant index with IHD and stroke (**A**: IHD; **B**: stroke). Age, gender, ethnicity, drinking status, smoking status, physical activity, education, BMI, TDI, diet score, sleep score, CRP, glucose, HbA1c, TG, cholesterol, fiber intake, B12 intake, hypertension, DM, and hyperlipidemia were adjusted.

**Figure 3:**
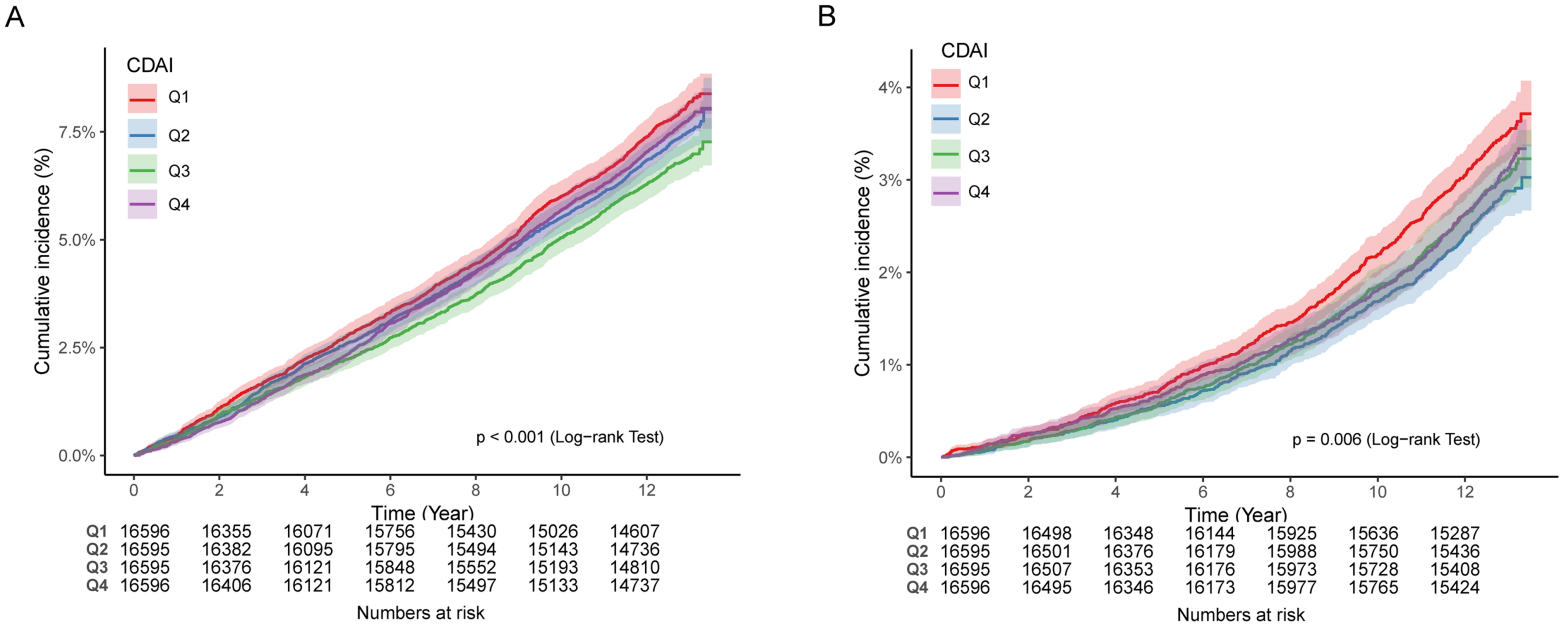
Kaplan–Meier curves for incident IHD **(A)** and stroke **(B)** according to CDAI quartiles.

After adjustment for multiple covariates, the CDAI showed no significant association with IHD (Table 2; Figure 2A). Compared with participants in the lowest quartile, those in the third quartile had a significantly reduced risk of IHD (HR 0.90; 95% CI 0.82–0.98), whereas no protective effect was observed in the highest quartile (HR 1.05; 95% CI 0.94–1.17). Consistent with these findings, Kaplan–Meier curves demonstrated that cumulative IHD incidence was highest among participants in Q1 and lowest in Q3, with Q4 showing an intermediate trajectory that converged toward higher risk over time. The separation between quartiles became apparent within the first few years of follow-up and persisted throughout, and the overall difference across groups was statistically significant (log-rank *p* < 0.001, Figure 3A).

**Table 2:** Association of composite dietary antioxidant index with IHD and stroke.

	Model 1 HR (95% CI)	Model 2 HR (95% CI)	Model 3 HR (95% CI)
IHD			
CDAI	0.96 (0.93, 0.99)	0.97 (0.94, 1.00)	1.01 (0.96, 1.06)
CDAI category			
Q 1	1.0	1.0	1.0
Q 2	0.88 (0.81, 0.95)	0.90 (0.83, 0.97)	0.96 (0.89, 1.05)
Q 3	0.80 (0.74, 0.87)	0.82 (0.76, 0.89)	0.90 (0.82, 0.98)
Q 4	0.91 (0.84, 0.98)	0.94 (0.87, 1.01)	1.05 (0.94, 1.17)
Stroke			
CDAI	0.96 (0.92, 1.00)	0.97 (0.93, 1.01)	1.00 (0.92, 1.08)
CDAI category			
Q 1	1.0	1.0	1.0
Q 2	0.76 (0.67, 0.86)	0.78 (0.69, 0.88)	0.82 (0.72, 0.94)
Q 3	0.81 (0.72, 0.91)	0.83 (0.74, 0.94)	0.88 (0.77, 1.02)
Q 4	0.83 (0.73, 0.93)	0.85 (0.76, 0.96)	0.90 (0.75, 1.07)

Model 1: Age, gender, and ethnicity were adjusted.

Model 2: Age, gender, ethnicity, drinking status, smoking status, and physical activity were adjusted.

Model 3: Age, gender, ethnicity, drinking status, smoking status, physical activity, education, BMI, tdi, diet score, sleep score, CRP, glucose, Hba1c, TG, Cholesterol, fibre intake, B12 intake, hypertension, DM, and hyperlipidemia were adjusted.

### Association between CDAI and stroke

For stroke, higher CDAI was associated with a lower risk in model 1, but the association attenuated and lost statistical significance in the fully adjusted model (Table 2, Figure 2B). A modest dose–response trend was observed, with participants in the second quartile showing a significantly reduced risk compared with the lowest quartile (HR 0.82; 95% CI 0.72–0.94). Associations in higher quartiles were not significant. Kaplan–Meier curves supported these findings, showing that cumulative incidence was highest in Q1 and lowest in Q2, with Q3 and Q4 following intermediate and overlapping trajectories (Figure 3B). The divergence between Q1 and Q2 was evident early during follow-up and remained stable thereafter, and the overall log-rank test confirmed significant differences across quartiles (log-rank *p* = 0.006, Figure 3B).

### Dose–response and threshold analyses

Restricted cubic spline models revealed significant nonlinear associations between CDAI and the risks of IHD and stroke (P for nonlinearity = 0.005 and 0.008, respectively; Figure 2). For IHD, the exposure–response relationship followed a U-shaped curve: the risk decreased sharply with rising CDAI values from the lowest levels up to approximately −0.30, after which the slope flattened and showed a slight upward tendency. Two-piecewise linear regression further confirmed this threshold effect, identifying an inflection point at CDAI = −0.30. Below the threshold, each 1-unit increase in CDAI was associated with an 11% reduction in IHD risk (HR 0.89, 95% CI 0.81–0.97; *p* = 0.010), whereas above the threshold, no significant association was observed (HR 1.05, 95% CI 1.00–1.11; *p* = 0.065) (Table 3).

**Table 3:** Threshold effect analysis of composite dietary antioxidant index with IHD and stroke using a two-piecewise linear regression model.

	Adjust HR (95% CI)	*p* value
IHD		
Fitting by standard linear model	1.01 (0.96, 1.06)	0.823
Fitting by two-piecewise linear model		
Inflection point	−0.30	
< −0.30	0.89 (0.81, 0.97)	0.010
> − 0.30	1.05 (1.00, 1.11)	0.065
Log-likelihood ratio	0.001	
Stroke		
Fitting by standard linear model	1.00 (0.92, 1.08)	0.954
Fitting by two-piecewise linear model		
Inflection point	−0.29	
< −0.29	0.82 (0.72, 0.95)	0.006
> − 0.29	1.07 (0.98, 1.17)	0.11
Log-likelihood ratio	0.001	

Age, gender, ethnicity, drinking status, smoking status, physical activity, education, BMI, tdi, diet score, sleep score, CRP, glucose, Hba1c, TG, Cholesterol, fibre intake, B12 intake, hypertension, DM, and hyperlipidemia were adjusted.

For stroke, the dose–response curve displayed a reverse J-shaped pattern, with risk decreasing as CDAI rose until around −0.29, beyond which the association plateaued. The two-piecewise linear model confirmed an inflection point at CDAI = −0.29. Below this point, each 1-unit increase in CDAI corresponded to an 18% lower risk of stroke (HR 0.82, 95% CI 0.72–0.95; *p* = 0.006), whereas above the threshold, no further benefit was observed (HR 1.07, 95% CI 0.98–1.17; *p* = 0.11) (Table 3). Likelihood ratio tests indicated that the two-piecewise linear models provided a better fit than the simple linear models for both IHD and stroke (*p* = 0.001), reinforcing the presence of nonlinear and threshold effects.

### Subgroup analyses

**Figure 4:**
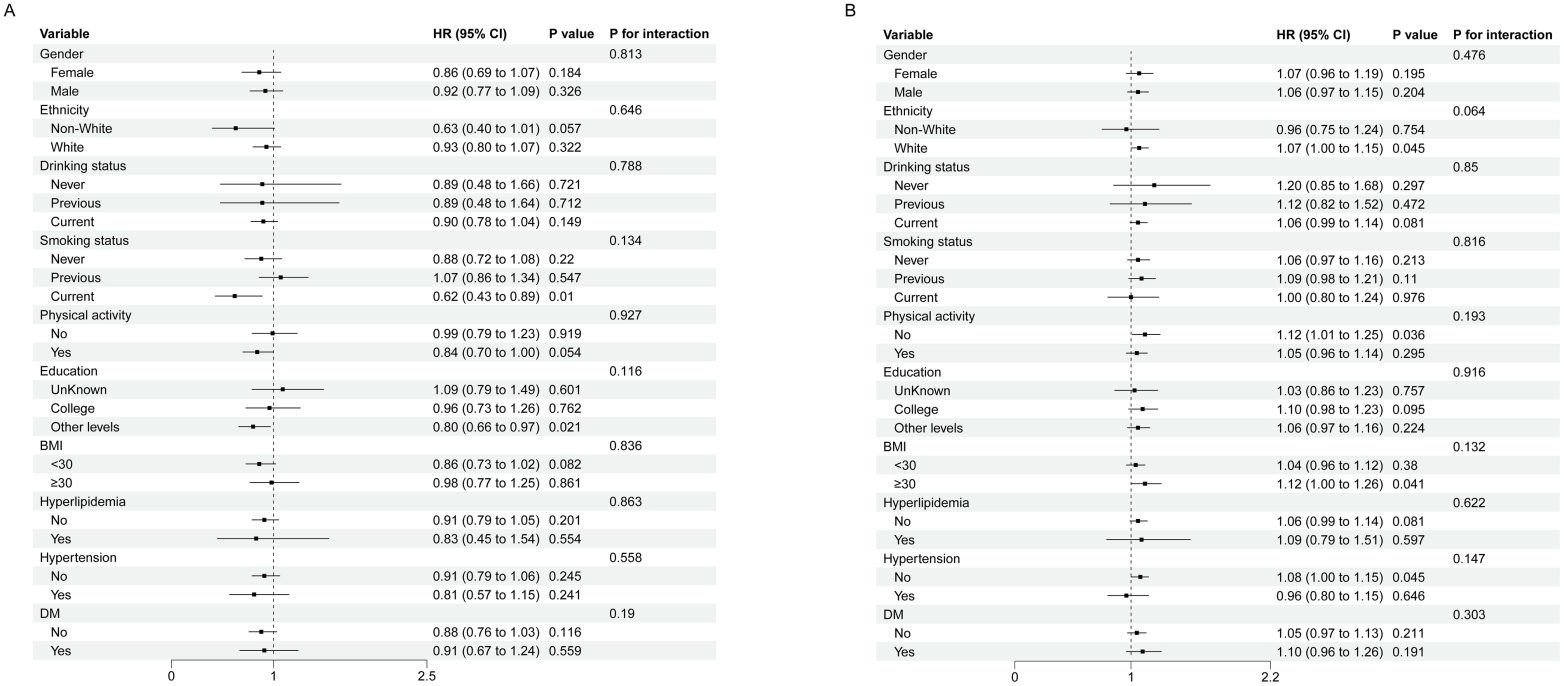
Subgroup analysis of association between composite dietary antioxidant index and IHD (**A**: CDAI < −0.30; **B**: CDAI > − 0.30).

**Figure 5:**
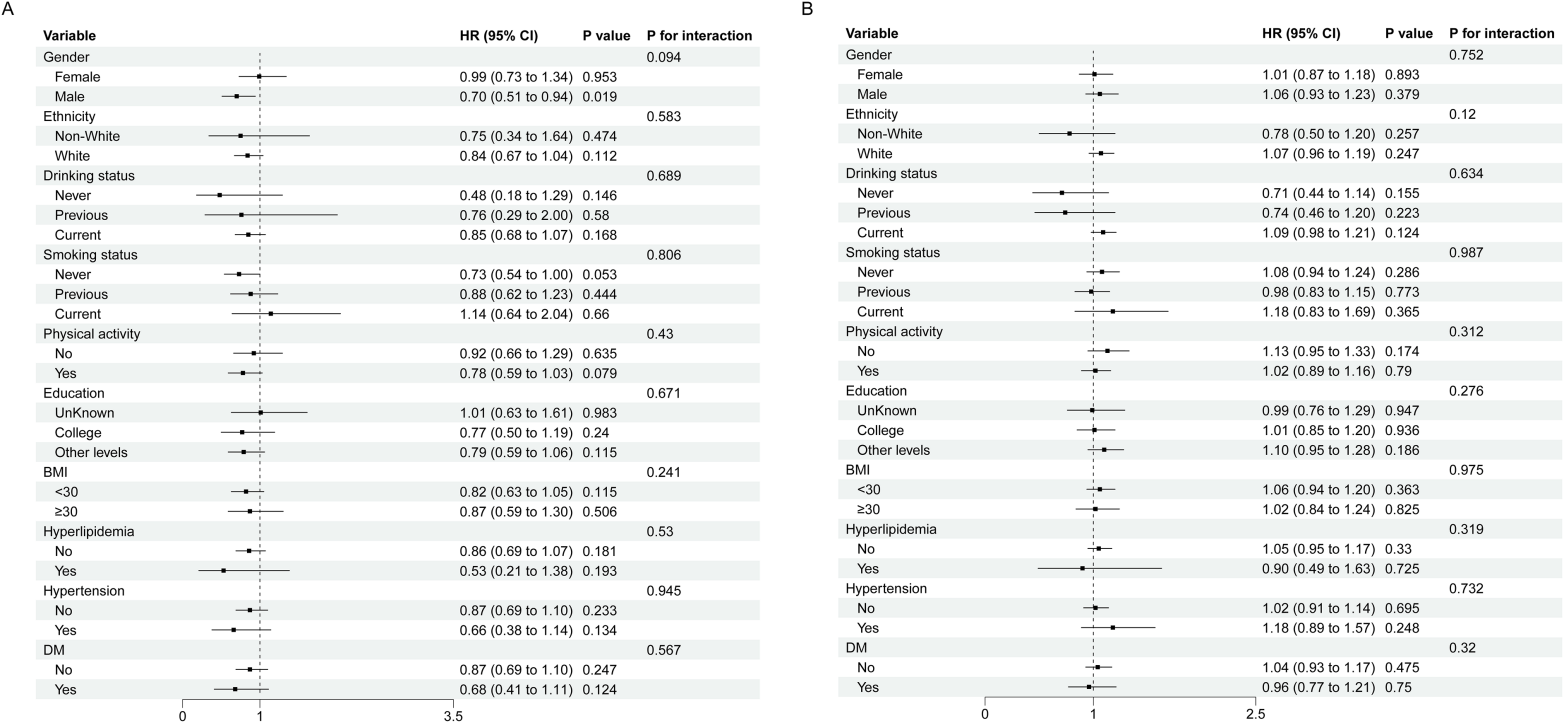
Subgroup analysis of association between composite dietary antioxidant index and stroke (**A**: CDAI < −0.29; **B**: CDAI > − 0.29).

Subgroup analyses were conducted to assess population differences in the association between CDAI and cardiovascular outcomes (Figures 4, 5). We performed interaction tests in groups defined by the inflection points to avoid statistical bias arising from overall non-linear result. For ischemic heart disease, the association between CDAI and risk was consistent across gender, education, ethnicity, lifestyle, and clinical subgroups, with no significant interactions (Figure 4). Similar results were observed for stroke, where the association showed no variation by demographic, behavioral, or clinical factors (Figure 5). These findings indicate that the associations between the CDAI and risks of IHD and stroke were stable across populations.

## Discussion

Using data from the UK Biobank cohort, this extensive prospective study evaluated the relationship between the composite dietary antioxidant index (CDAI) and the incidence of ischemic heart disease (IHD) as well as stroke. Several key findings emerged. First, higher CDAI values were associated with a lower risk of IHD and stroke within a specific range, although these associations attenuated and were no longer statistically significant after full adjustment. Although the observed associations were modest in magnitude, even small relative risk reductions at the population level may translate into substantial public health benefits, particularly given the high global burden of cardiovascular diseases. Second, restricted cubic spline analyses revealed significant nonlinear dose–response relationships, with threshold effects at CDAI values of −0.30 for IHD and −0.29 for stroke. Below these thresholds, incremental increases in CDAI were associated with substantially reduced disease risk, while no additional benefit was observed beyond the inflection points. Third, subgroup analyses suggested that these associations were generally consistent across any subgroup. Importantly, the consistency of these associations across demographic and lifestyle subgroups reinforces their relevance for population-wide dietary guidance and public health practice.

Our findings extend the current literature on dietary antioxidants and cardiovascular health. Prior studies have yielded inconsistent results when examining single nutrients, such as vitamin C, vitamin E, or carotenoids, likely due to collinearity among dietary factors and the synergistic actions of multiple antioxidants (8, 21–23). By employing the CDAI, which integrates multiple dietary antioxidants, this study provides a more comprehensive assessment of overall antioxidant exposure than single-nutrient approaches. Recent investigations, such as those by Teng et al. in NHANES and Lin et al. in Chinese adults, reported inverse nonlinear associations between CDAI and stroke or atherosclerotic cardiovascular disease, while Hu et al. demonstrated a protective association between CDAI and mortality in patients with established CVD (18). Our findings align with these reports. Importantly, the identification of threshold effects represents a novel contribution, emphasizing the need to consider nonlinear relationships in nutritional epidemiology.

The nonlinear patterns observed deserve particular attention. For IHD, the protective association was evident up to a CDAI of −0.30, beyond which the relationship plateaued and even showed a modest upward trend. Although a slight increase in risk was observed at higher CDAI levels, this trend was not statistically significant and should therefore be interpreted with caution. Causal inferences cannot be established due to the observational nature of the study, and the findings should be interpreted as associations rather than evidence of causality. For stroke, the inverse association followed a reverse J-shaped curve, again suggesting diminishing benefits above a certain intake level. These findings imply that antioxidant intake may exert its greatest preventive effect among individuals with low baseline exposure, while excessive intake may confer little additional advantage. However, the observed nonlinear associations may also be influenced by residual confounding or measurement error, and should be interpreted cautiously in the context of potential residual confounding and measurement error. Mechanistically, while moderate antioxidant intake helps to neutralize excess reactive oxygen species (ROS) and mitigate oxidative stress, supraphysiological doses may disturb the redox balance. Experimental and clinical studies suggest that excessive antioxidant supplementation may disrupt redox balance and provide limited or even adverse effects under certain conditions (24–28). These findings support the possibility that the cardiovascular benefits of antioxidants may plateau beyond a moderate intake range. Moreover, excessive antioxidant intake may interfere with endogenous adaptive responses. Oxidative stress at low-to-moderate levels functions as a signaling mechanism that induces protective pathways, such as Nrf2-mediated upregulation of antioxidant genes (29). Over-supplementation may blunt this adaptive hormetic response, weakening the body’s intrinsic defense system (30). Furthermore, excessive intake of single antioxidants may disrupt the natural synergy among dietary micronutrients, altering metabolic pathways in ways that counteract expected benefits. Dietary antioxidants have been shown to modulate inflammatory pathways and redox balance, further supporting their role in cardiovascular health (31–33). These mechanisms align with our findings of threshold effects in both IHD and stroke, where protective associations were restricted to lower CDAI ranges. From a clinical and public health perspective, the identified inflection points (−0.30 and −0.29) should not be interpreted as strict cut-off values, but rather as indicative thresholds reflecting a transition from insufficient to adequate antioxidant intake. Given that CDAI is a standardized composite score, these values likely correspond to a relative improvement from suboptimal dietary patterns toward more balanced, antioxidant-rich diets. Importantly, these findings suggest that the greatest cardiovascular benefit may be achieved by improving antioxidant intake among individuals with low baseline exposure, rather than promoting excessive intake. This has practical implications for dietary guidance, emphasizing achievable dietary modifications (e.g., increased consumption of fruits, vegetables, and whole grains) rather than high-dose antioxidant supplementation. They are also consistent with previous meta-analyses of randomized controlled trials showing that antioxidant supplementation does not reduce—and may even increase—cardiovascular risk when given in high doses (34). Taken together, the results highlight the importance of obtaining antioxidants from balanced dietary sources rather than indiscriminate supplementation. This interpretation also helps bridge the gap between epidemiological findings and actionable dietary recommendations.

This study has several strengths. The UK Biobank provides a uniquely large sample size, detailed dietary data through repeated 24-h recalls, long follow-up, and extensive covariate adjustment, allowing robust estimation of associations. The use of the CDAI enabled a comprehensive assessment of dietary antioxidant exposure while minimizing measurement error through energy adjustment and standardization. Furthermore, dose–response and threshold analyses offered novel insights into the nonlinear nature of these associations. Moreover, the ability to characterize dose–response thresholds may contribute to refining evidence-based dietary recommendations aimed at CVD prevention.

Nonetheless, several limitations should be acknowledged. First, dietary intake was assessed using self-reported 24-h recalls, which are subject to recall bias, misreporting, and measurement error. Although repeated assessments were used when available to better approximate habitual intake, changes in dietary patterns over time cannot be fully captured. In addition, the CDAI includes only a limited number of antioxidant nutrients and does not account for other dietary components that may influence oxidative stress, potentially leading to incomplete assessment of overall antioxidant exposure. Second, the CDAI does not capture antioxidant intake from supplements, which may result in underestimation and potential exposure misclassification, particularly among individuals with high supplement use. However, given that supplement use is not universal and varies across populations, the overall impact on the observed associations is likely limited. Moreover, focusing on food-derived antioxidants may better reflect real-world dietary patterns, and prior studies suggest differential biological effects between dietary and supplemental antioxidants. Third, residual confounding cannot be entirely excluded despite extensive adjustment. Some factors, such as genetic predisposition, healthcare access, detailed dietary components, and other lifestyle variables, may not have been fully captured and measurement error in self-reported covariates may have resulted in incomplete adjustment. Additionally, categorizing CDAI into quartiles may have reduced statistical power and obscured more complex dose–response relationships. Fourth, the UK Biobank cohort is characterized by a “healthy volunteer” selection bias and represents a relatively healthy and socioeconomically advantaged population, which may limit generalizability. Therefore, caution is warranted when extrapolating these findings to more diverse or high-risk populations.

Finally, the observational design precludes causal inference, although the prospective design reduces the likelihood of reverse causation.

## Conclusion

In conclusion, this prospective cohort study suggested that higher dietary antioxidant intake, as reflected by CDAI, was associated with reduced risks of IHD and stroke, particularly below identified intake thresholds. The identification of nonlinear threshold effects provides new insights into the role of antioxidants in cardiovascular prevention, suggesting that “more is not always better.” These findings underscore the relevance of balanced, antioxidant-rich dietary patterns—rather than high-dose supplementation—as a feasible and sustainable public health strategy for cardiovascular disease prevention. Taken together, these findings support ongoing efforts to integrate nutrition-focused strategies into cardiovascular prevention frameworks, emphasizing food-based approaches that are equitable, sustainable, and scalable at the population level.

## Data Availability

The original contributions presented in the study are included in the article/supplementary material, further inquiries can be directed to the corresponding authors.

## Footnotes

1
https://www.ukbiobank.ac.uk/enable-your-research/about-uk-biobank
